# Effectiveness of Integration and Re-Integration into Work Strategies for Persons with Chronic Conditions: A Systematic Review of European Strategies

**DOI:** 10.3390/ijerph15030552

**Published:** 2018-03-19

**Authors:** Carla Sabariego, Michaela Coenen, Elizabeth Ito, Klemens Fheodoroff, Chiara Scaratti, Matilde Leonardi, Anastasia Vlachou, Panayiota Stavroussi, Valentina Brecelj, Dare S. Kovačič, Eva Esteban

**Affiliations:** 1 Research Unit for Biopsychosocial Health, Department of Medical Information Processing, Biometry and Epidemiology (IBE), Ludwig-Maximilians-Universität (LMU), 81377 Munich, Germany; michaela.coenen@med.lmu.de (M.C.); elizabeth.ito.88@gmail.com (E.I.); eva.esteban@med.lmu.de (E.E.); 2 Neurorehabilitation KABEG-Gailtal-Klinik, 9620 Hermagor, Austria; klemens.fheodoroff@kabeg.at; 3 Neurology, Public Health and Disability Unit; Neurological Institute C. Besta IRCCS Foundation, 20133 Milan, Italy; chiara.scaratti@istituto-besta.it (C.S.); matilde.leonardi@istituto-besta.it (M.L.); 4 Department of Special Education, University of Thessaly, 38221 Volos, Greece; anavlachou@uth.gr (A.V.); stavrusi@uth.gr (P.S.); 5 Development Centre for Vocational Rehabilitation, University Rehabilitation Institute Republic of Slovenia, 1000 Ljubljana, Slovenia; valentina.brecelj@ir-rs.si (V.B.); dare.kovacic@ir-rs.si (D.S.K.)

**Keywords:** review, effectiveness, chronic disease, musculoskeletal diseases, disability, employment, return to work, sick leave, Europe

## Abstract

Due to low employment rates associated to chronic conditions in Europe, it is essential to foster effective integration and re-integration into work strategies. The objective of this systematic review is to summarize the evidence on the effectiveness of strategies for integration and re-integration to work for persons with chronic diseases or with musculoskeletal disorders, implemented in Europe in the past five years. A systematic search was conducted in MedLine, PsycINFO, CDR-HTA, CDR-DARE and Cochrane Systematic Reviews. Overall, 32 relevant publications were identified. Of these, 21 were considered eligible after a methodological assessment and included. Positive changes in employment status, return to work and sick leave outcomes were achieved with graded sickness-absence certificates, part-time sick leave, early ergonomic interventions for back pain, disability evaluation followed by information and advice, and with multidisciplinary, coordinated and tailored return to work interventions. Additionally, a positive association between the co-existence of active labour market policies to promote employment and passive support measures (e.g., pensions or benefits) and the probability of finding a job was observed. Research on the evaluation of the effectiveness of strategies targeting integration and re-integration into work for persons with chronic health conditions needs, however, to be improved and strengthened.

## 1. Introduction 

People with chronic diseases face a variety of important problems in performing their every-day lives and in participating in society, being work one of the major areas affected [1,2]. Chronic diseases, also known as non-communicable diseases (NCDs), are defined by the World Health Organization (WHO) as diseases that are not passed from person to person, that have generally a long duration and slow progression [3]. They affect people of all ages and a significant proportion of persons in working age are limited and restricted in their ability to obtain and maintain employment, especially when no accommodations are in place [4]. Moreover, chronic diseases, such as low back pain, migraine, diabetes and depression, have in common that their peaks in disease onset are in the most productive years of adults’ working lives [5]. Indeed, available statistics show that persons with chronic diseases have lower employment rates [6] and more difficulties in finding and keeping a job compared to people without such disease [7].

Especially in Europe, which has an increasing ageing population, there is a growing number of persons with chronic diseases in working age and their number will steadily increase in the next decades [8]. According to the International Diabetes Federation (IDF) Atlas of Diabetes, for example, the prevalence of diabetes will increase from 58 million in 2017 to 66.7 million in 2045 in the European adult population [9]. This increasing number will raise the individual and economic burden for the persons themselves, their families, as well as for the societies they are living in. For societies and stakeholders at national as well as European levels it will be essential to deal with this challenge. For persons with diabetes alone the health expenditure per year in Europe was about 166 billion USD in 2017 [9]. To face this challenge, WHO has called for action with its Global action plan for the prevention and control of non-communicable diseases in 2013–2020 [10].

Due to the societal and economic burden of low employment rates associated to chronic conditions in Europe, it is essential to foster effective integration and re-integration into work strategies. There is a wide range of general and disease-specific strategies implemented at regional and national level in European countries [11,12]. These strategies range from implementing incentive-based systems at national levels to the implementation of tailored interventions and case management approaches (see also overview of Oortwijn and colleagues [13]). For instance the concept of Flexicurity—in which an optimal combination of active labour market policies and passive measures to maintain social security, such as disability benefits, is targeted—proved to be promising where it has been tested, such as in Italy [14], and could be potentially recommended to be implemented in other European countries as well. An open question, however, is to what extent existing strategies are indeed effective. To decide on which work integration and re-integration strategies to implement, European countries would therefore benefit from an overview of evidence-based strategies already implemented at national as well as at regional and institutional levels in Europe.

The EU-funded Participation to Healthy Workplaces and Inclusive Strategies in the Work Sector project (PATHWAYS; www.path-ways.eu) aims to identify strategies of integration and reintegration to work for persons with chronic diseases in Europe, to evaluate their effectiveness and to assess the specific employment-related needs of these persons. To evaluate the evidence on the effectiveness, a comprehensive systematic review was carried out in PATHWAYS for a wide range of chronic conditions and three major groups of publications were identified: (1) studies focusing on persons with mental disorders; (2) studies focusing on persons with musculoskeletal disorders; and (3) studies focusing on persons with chronic conditions in general, i.e., studies in which specific conditions were not further specified or in which results for different conditions were reported together. This third group also included persons with disability, as usually the majority of people who receive disability benefits in Europe have chronic diseases and experience significant levels of disability in daily life [13]. Studies approaching disability from an impairment perspective (sensorial, physical or intellectual) were not considered. This paper is part of this large review and focuses on groups 2 and 3. The objective of this paper is therefore to systematically review the evidence on the effectiveness of strategies for integration and re-integration to work for persons with chronic diseases and for persons with musculoskeletal disorders, implemented in European countries in the past five years.

## 2. Materials and Methods

We performed a systematic literature review in the scope of the EC-funded PATHWAYS (Participation to Healthy Workplaces and Inclusive Strategies in the Work Sector; www.path-ways.eu) project. The PATHWAYS Consortium is made up of partners from ten European countries: Austria, Belgium, Czech Republic, Germany, Greece, Italy, Norway, Poland, Slovenia and Spain. It is important to stress that the searches of the systematic literature review run within the scope of the PATHWAYS project focused on a wide range of diseases, such as mental disorders, musculoskeletal disorders, cancer, neurological, metabolic, respiratory and cardiovascular diseases and different study designs.

### 2.1. Search Strategy for the Complete PATHWAYS Review

A systematic search was conducted using the databases: MedLine, PsycINFO, CDR-HTA, CDR-DARE and Cochrane Systematic Reviews. All search strategies are described in the Supplementary Material. Searches were run in April 2016. Additionally, reference lists of included papers and of studies included in 30 systematic reviews and meta-analyses published between 2011 and April 2016 were also reviewed for papers not identified in the electronic search.

### 2.2. Selection Criteria for the Complete PATHWAYS Review

Studies were included if they:

(a) had been published between January 2011 and April 2016;
(b) were published in English;
(c) were intervention studies, namely randomized trials, non-randomized controlled trials, non-controlled pre-post intervention studies;
(d) were observational studies, namely cohort studies, case-control studies, cross-sectional studies, descriptive longitudinal studies,
(e) were qualitative studies;
(f) had been carried out in the 28 countries of the European Union, in Norway, Lichtenstein, Iceland or Switzerland, or in non-European countries with western lifestyle: Canada, United States of America, Australia;
(g) reported on effectiveness regarding at least one of the following work outcomes:
  (1) employment status (employed/unemployed);
  (2) return to work;
  (3) absenteeism (sick leave);
  (4) maintain a job;
  (5) obtain a job.
(h) investigated variables potentially affecting effectiveness (e.g., views and experiences of involved persons with a given strategy).

Regarding the target population, studies were included if they focused on the working age population, namely persons aged 16 to 65 years. Regarding health conditions, studies were included if they focused on:

(a) persons with chronic diseases in general, i.e., specific conditions are not further specified in the studies or results for different conditions are reported together, and persons with disability in general. Persons with disability were included as usually the majority of people who receive disability benefits have chronic diseases and experience significant levels of disability in daily life [13];
(b) the following disease groups: mental disorders, musculoskeletal disorders, cancer, neurological, metabolic, respiratory and cardiovascular diseases;
(c) the following specific diseases: depression, back and neck pain, migraine, diabetes mellitus, chronic obstructive pulmonary disease and ischemic heart disease.

Studies were excluded if they:

(a) included participants with mainly other chronic diseases as the ones defined above and only pooled results were reported;
(b) included participants aged <16 or >65 years;
(c) were case report/case series, psychometric studies, letters, comments, editorials, overviews without empirical primary or secondary data, reviews (systematic and non-systematic reviews, health technology assessments) and meta-analyses, protocols, studies reporting exclusively on design or baseline data;
(d) consider neither effectiveness outcomes, for example, studies reporting only on costs resulting from the implementation of strategies nor variables potentially affecting effectiveness;
(e) did not focus on a concrete strategy or group of strategies, for example, studies focusing on factors facilitating return to work after sick leave in general;
(f) were published in other languages than English;
(g) were published before 2011;
(h) had no abstract available.

### 2.3. Eligibility Assessment for the Complete PATHWAYS Review

Abstracts retrieved from the searches were checked using the pre-defined inclusion and exclusion criteria by trained reviewers. Approximately 30% of the references were double checked independently by a second reviewer. Full versions of papers considered eligible were retrieved and examined by two researchers.

### 2.4. Data Extraction and Data Synthesis for the Complete PATHWAYS Review

The following data of included papers was extracted by one reviewer and checked by a second researcher: objectives of the study; study design; study population; intervention; assessment time points; work outcomes and their operationalization; and results.

### 2.5. Methodological Assessment for the Complete PATHWAYS Review

Included studies were independently assessed by two researchers using the quality appraisal checklist for quantitative intervention studies and the checklist for quantitative studies reporting correlations and associations published by the National Institute for Health and Care Excellence (NICE) in its methodological guide to develop public health guidance [15]. In the assessment, two groups have been defined based on shortcomings of either the study or of the corresponding publication:

(a) shortcomings are unlikely to change study’s conclusions regarding the outcomes of interest;
(b) shortcomings are likely or very likely to change study’s conclusions regarding outcomes of interest.

Only studies classified as “a” have been included in the synthesis.

Regarding effectiveness, we answered the question on whether data supported the effectiveness of the strategy with four categories:

- *Yes*. Yes was selected if estimates for relevant work outcomes had an adequate *p*-value, usually <0.05, or if the confidence interval for the estimate excluded the no-effect value (e.g., the value 1 was not included in the confidence interval of reported odds ratio);
- *Unclear*. Unclear was selected if the precision of the effect estimate was not reported, results were inconsistent or difficult to interpret (e.g., statistically non-significant but large estimates in subgroup analyses);
- *No*. No was selected if data did not support the presence of an effect of the intervention on relevant work outcomes.

The searches carried out in the PATHWAYS project had a very broad scope and were developed to identify quantitative and qualitative papers, and a comprehensive population, as described above.

This manuscript focuses, however, solely on quantitative studies evaluating the effectiveness of interventions carried out in European countries with persons with chronic diseases in general (specific conditions are not further specified in the studies or results for different conditions are reported together) or persons with disability (because, as described above, usually the majority of people who receive disability benefits have chronic diseases) and for persons with musculoskeletal disorders. Results for mental disorders and results of qualitative papers focusing on factors that affect effectiveness are reported in other publications of this special issue [16,17].

Results are reported following the PRISMA statement [18]. Data synthesis will be presented by kind of interventions (policy, system or service) and results will be summarized into three headings: “studies reporting positive change”, “studies with unclear results” and “studies reporting no change”. If possible, a quantitative comparison of studies will be conducted.

## 3. Results

### 3.1. Study Selection and Characteristics of Studies

**Figure 1:**
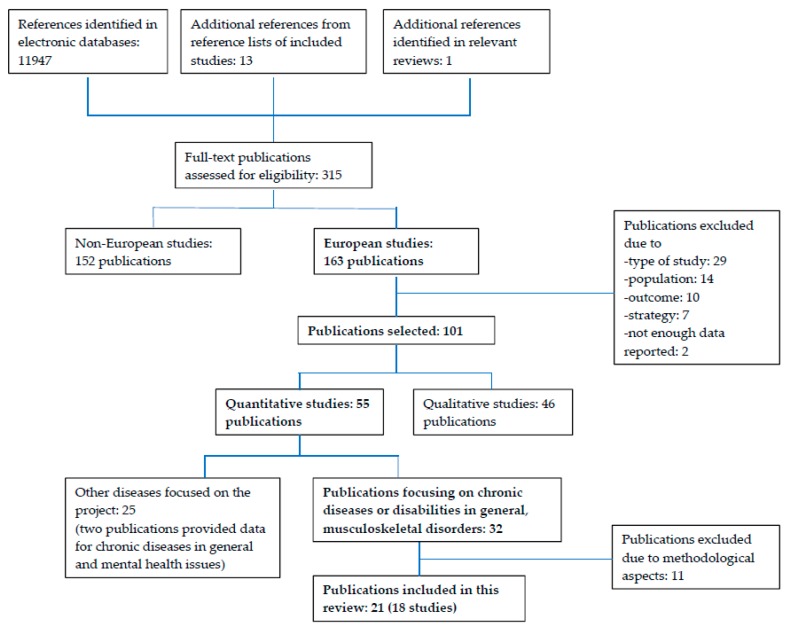
Flow chart of the systematic review carried out in the PATHWAYS project. Boxes in bold highlight the flow chart of the present review with its focus on quantitative papers evaluating strategies for persons with chronic conditions in general, disability or musculoskeletal conditions.

A total of 32 publications were identified which evaluated strategies for persons with chronic diseases and disability in general (*n* = 15) or musculoskeletal disorders (*n* = 17). Of these, 21 publications were considered to be reliable after the methodological assessment, i.e., to have shortcomings in the study design or in the reporting of the study that are unlikely to change the study’s conclusions, and included in the present systematic review (Table 1). The flow chart of the study selection process in PATHWAYS is presented in Figure 1. Boxes printed in bold highlight the relevant numbers for the present review with its focus on quantitative papers carried out in European countries and evaluating strategies for persons with chronic conditions and disability in general or musculoskeletal disorders.

The included 21 publications reported on 18 studies. The reason for having more publications than studies is that results of single studies have been reported in more than one scientific article, for instance results for 12 and 24 months follow-ups or secondary data analyses focusing on specific groups were reported and published separately. From the 18 studies, 13 were conducted in Nordic countries: four in Denmark, five in Finland, two in Norway and two in Sweden (Table 1). The remaining five studies were conducted in Italy, Spain, Switzerland, the Netherlands and Belgium. Eight studies were randomized controlled trials, one was a controlled trial, eight studies were cross-sectional or cohort studies based on register data and one study used two methods to explore its research question (i.e., RCT and cohort study [20]). Nine studies evaluated strategies for persons with musculoskeletal disorders while nine focused either on chronic diseases in general or on persons with disability. Finally, only two studies evaluated policy strategies and the majority of the studies, altogether ten, evaluated services, as described below. A quantitative summary of the effect of reported interventions was not considered appropriate because of the important methodological differences between studies, and was therefore not carried out.

### 3.2. Interventions for Chronic Diseases and Disability in General

Interventions for chronic diseases and disability in general were mostly targeted at employed persons on sick leave (Table 2). Four studies [22,23,24,25] evaluated Part-Time Sick Leave (PTSL) in three countries: Finland, Norway and Sweden. PSTL may be seen as a complex intervention that requires an initial joint decision made by the individual, the employer, the physician, and the social insurance administrator, and actions and decisions on the part of the employee, colleagues and employer to adjust both work time and work demands. The results of these four studies supported the facilitating role of PTSL regarding work participation. Additionally, Halonen (2016) showed a positive impact of legislative changes obligating notification of prolonged sickness absence and assessment of remaining work (“30-60-90 day rule”). Two studies evaluated multidisciplinary interventions [20,21] but only one was effective [21]. This study had more comparable groups and carried out the analyses by center, observing that the municipality with more severe cases achieved better results. The disability support benefit evaluated in Spain [19] was characterized by the provision of financial support or benefits, no involvement of workplace or employers and the possibility of PTSL (Table 2). Additionally, the person was required to take a different job to the one he or she had before the benefits. It is frequently hypothesized that such benefits would disincentive people from working. However, a negative association between the disability benefit and employment was observed in the study only for persons with the mildest levels of disability. An intervention targeting policy changes evaluated Flexicurity in Italy [14], i.e., the co-existence of active labour market policies and passive measures such as benefits, and showed positive and promising results.

### 3.3. Interventions for Musculoskeletal Disorders

Interventions for musculoskeletal disorders were with the exception of two studies [27,31] targeted at employed persons, most of them on sick leave (Table 3). Five publications out of 12 reported on evaluations of three multidisciplinary interventions. As shown in Table 3, although all involved multidisciplinary teams, the interventions differed considerably regarding other aspects. For instance, two included financial support or benefits, and two considered the workplace or involved employers. The two effective or partly effective interventions [28,30,31] had two characteristics that the other interventions lacked: the provision of financial support or benefits and the involvement of workplace or employers. It is important to note, however, that one non-effective intervention [27] was tested in a very small sample (*n* = 45) and that the power of the study was low to capture group differences. The two interventions focusing on either education [32] or work-focused counselling [33] were quite different regarding scope (Table 3). While providing information and advice during a disability evaluation effectively reduced sick leave [32], counselling by an occupational physician had a significant effect for self-reports of sick leave for more than 8 weeks and for cumulated sick leave days but no effect when register data on sick leave of more than 2 weeks due to all causes was considered (Table 1). Two studies evaluating PTSL [37,38] showed positive effects and results that are consistent with the ones for persons with chronic diseases in general and disability. Finally, an ergonomic intervention [36] was also effective in reducing sickness absence due to any MSD in the long-run (Table 1).

### 3.4. Interventions by Level

#### 3.4.1. Policy Level

Policies are binding and non-binding legislative frameworks, provisions and policy approaches that set a course or a principle of action at local, regional, national or international level, for instance anti-discrimination law [11].

In Italy, Agovino and Rapposelli [14] investigated whether the co-existence of (a) active labour market policies to promote the employment of people with disability and (b) passive measures to support people with disability (e.g., disability pensions) was positively related to the probability of obtaining work, i.e., whether the combination of passive and active strategies—a core concept behind the Flexicurity strategy—could increase employability of persons with disability. More specifically, Agovino and Rapposelli calculated different indexes of “Flexicurity” giving different weights to existing active and passive measures in Italy—the estimate for active measures was the amount of Regional Fund for Employment of People with Disabilities assigned to a region while the estimate for existing passive measures was the amount of percipients of civilian disability pensions in working age—and assigned each Italian region a value. They explored, whether there was an association between the different indexes and the amount of people with a disability searching a job who obtained a job. After controlling for context variables, their results support a positive effect on employment of the combination of active and passive measures, an effect not given when active and passive measures are considered separately.

In Finland, one study evaluated the impact of legislation changes. Halonen [26] used register data to evaluate how legislative changes obligating notification of prolonged sickness absence and assessment of remaining work (“30-60-90 day rule”) affected return to work of public-sector employees with permanent job contract and on sick-leave for at least 30 calendar days. Workers who had been 60 days on sick leave returned to work earlier after introduction of the law but the effect was larger for women than for men and for the low than the high job status group.

#### 3.4.2. System Level

Strategies at system level include supports, programmes or schemes (including financial support) aimed at supporting unemployed and inactive persons in obtaining or returning to paid employment; supporting employed persons in remaining at work; supporting employers and employment services in facilitating the participation of persons with chronic diseases in paid employment, for instance through supported employment programs [11].

##### Disability Support Benefit for Persons with Long-Lasting Disability

A disability support benefit, defined as an economic benefit for individuals affected by a pathologic or traumatic process causing long-lasting disability, was evaluated in 2014 in Spain by Lopez Frutos and colleagues [19]. Two groups were compared in this cross-sectional study using register-based data: a large group of persons with a certificate of disability and disability support benefit (*n* = 27,660) and a control group of persons with the certificate of disability but no benefit (*n* = 19,976). The sample included persons who had a certificate of disability in 2008, 2009 or 2010. Using employment status as the outcome of interest, receiving a benefit had a significant negative direct effect on the probability of working for individuals on the disability threshold (disability level of 33–44%) and no statistically effect for individuals with a higher degree of disability (more than 45%). It is important to stress that Spain has been facing a rising unemployment rate since the global financial crisis in 2008, which overlaps with the time frame of this study: from 8% in June 2007 to a maximum rate of 27.2% in March 2013 (National Institute of Statistics).

##### Part-Time Sick Leave (PTSL)

Part-Time Sick Leave (PTSL) or PTSL benefits are primarily a system strategy that allows reduction in the contracted working hours or changes in the work tasks, while often compensating the worker for the resulting reduction in income.

##### Studies Reporting Positive Change

All studies reporting on PTSL have been carried out in Nordic countries. Kausto and colleagues [24] evaluated PTSL in Finland using a register-based cohort study and including persons with musculoskeletal disorders, among other diagnoses, who were full-time employed and on sick leave for at least 60 days. Persons in PTSL (*n* = 1047, 71.1% females) and persons in full sick leave (*n* = 28,380, 53% females) were compared and followed for at least 12 months. PTSL was associated with increased use of partial disability pension—an indicator of retaining a job despite impaired work ability—and decreased use of full disability pension—an indicator of leaving the labour market—in persons with musculoskeletal disorders, being the effect stronger for men. Overall results suggest enhanced work retention after PTSL.

Kausto [25] evaluated partial sick leave in Finland using register data and a cohort of persons people with chronic conditions, who were employed and on sick leave for at least for 60 days, observed for 12 months. Work participation decreased less when people received partial sick leave, with a larger effect for persons aged 45 to 65. After matching the sample, the effect was stronger and observed for all ages.

Viikari-Juntura evaluated PTSL for persons with musculoskeletal disorders in Finland [37] but used an RCT, a 12 months follow-up and relatively small samples of mostly women (97%) in PTSL (*n* = 31) and in full sick leave (*n* = 31) to evaluate the effectiveness of PTSL on time to sustained return to work—defined as working without recurrent sick leave—for ≥2 weeks and for ≥4 weeks. Findings suggest better work participation outcomes in the PTSL group, who achieved sooner return to work that sustained for at least 4 weeks and showed lower sick absence. It is important to stress that the samples in the study were quite healthy.

Markussen and colleagues evaluated in Norway [23] a graded sickness-absence certificate using a register-based cohort study and focusing on persons with chronic diseases or disability who were on long-term sick leave (at least 8 weeks). Large samples of persons with either graded sickness-absence certificate before the end of week 8 (*n* = 77,655) and persons with non-graded absence certificate (*n* = 261,596) were compared for two years. Graded sickness-absence certificate absence led to lower sick leave durations, less subsequent social security dependency, and higher employment propensities. It is important to stress that formal regulations encouraging employees, employers, and physicians to use the system are in place in Norway.

Andren et al. [38] examined in a Swedish cohort study if it is beneficial for individuals on sick leave due to musculoskeletal disorders to be on PTSL compared to full-time sick leave, looking for return to work with full recovery of lost work capacity as (primary) outcome. A sample of 1170 employees from the register database of the Social Insurance Agency of Sweden was used. PTSL was a significant and consistent predictor of return to work with full recovery in the model used. The study had a relatively long follow up of approximately one year.

Høgelund and colleagues [22] have evaluated PTSL in Denmark using a register-based cohort study. Persons with health problems in general (*n* = 638, 45% females), who were employed and on PTSL for more than eight weeks were compared to comparable persons on full-time sick leave for approximately one year and a half. PTSL significantly reduced the duration of sick leave for employees with other health conditions.

### 3.5. Service Level

Services strategies encompass activities by private or public entities aimed at assisting jobseekers in finding employment as well as social services that directly or indirectly contribute to the employability of persons with chronic diseases [11].

Work-Focused Strategies

Studies Reporting Positive Change

Shiri et al. [36] evaluated in Finland an early ergonomic intervention for employed persons, mostly women (87.3%) with upper-extremity back pain. Within the intervention, the physician contacts the employer after clinical examination, and a visit by the occupational physiotherapist is scheduled. The workplace is assessed and possible changes to achieve an ergonomic improvement are discussed with the employee and supervisor. Using a RCT with a 12 months follow-up, authors compared 91 persons receiving this intervention with a group of 86 persons receiving standard medical care. Results suggest that the early ergonomic intervention reduces sickness absence due to any musculoskeletal disorders in the long term (4- to 12-month period). The number of nurse-prescribed days in sick absence due to any musculoskeletal disorder was significantly lower in the intervention group but not the number of sickness absences prescribed by physicians and nurses. Subgroup analyses showed that subjects exposed to work-related physical load factors especially benefitted from the intervention.

Studies with Unclear Results

Jensen et al. [33] evaluated in Denmark a strategy addressing workplace barriers and physical activity, as part of an outpatient treatment for persons with low back pain. This strategy included counselling by an occupational physician, aiming at removing experienced workplace barriers as well as at enhancing physical activity of moderate intensity, pain, functioning and sick leave after 3 months. Two counselling sessions were integrated in low back pain secondary care. In their RCT with a 3 months follow-up, colleagues compared 150 (approx. 51% females) receiving this intervention with 150 persons (approx. 59% females) receiving usual care. Usual care typically consisted of a brief instruction in exercises, or readmission to a general practitioner for further contact with a physiotherapist or chiropractic treatment. All participants of the study had low back pain and were employed but expressed concerns about the ability to maintain their current job. The intervention had a significant effect for self-reports of both sick leave longer than 8 weeks and cumulated sick leave days due to low back pain. However, when researchers looked at register data on sick leave longer than 2 weeks due to all causes, there was no significant difference between the groups.

Studies Reporting No Change

In Norway, Myhre and colleagues [34] evaluated the effectiveness of additional work-focused intervention to multidisciplinary intervention. In the work-focused intervention a case manager analysed together with the patient work and return to work difficulties, developed with the person a return to work schedule, discussed relevant issues for a meeting with the employer and if sick-leave compensation was an issue, contacted municipal social services. Participants were persons with neck and back pain, who were employed but on sick leave for at least 4 and at most 12 weeks. Persons receiving multidisciplinary intervention (brief or comprehensive) (*n* = 202, 49% females) were compared to persons receiving multidisciplinary intervention and the additional work-focused intervention (*n* = 203, 44% females) in an RCT with 12 months follow-up. Adding a work-focused intervention didn’t increase the effect of multidisciplinary care in decreasing time to return to work, except for subjects older than 41 years. The additional intervention had no effect on the total number of subjects returning to work but was not inferior to interventions that focus on physical activity and pain. Marchand et al. [35] carried out a secondary analysis of Myhre’s RCT to explore secondary clinical outcomes and the influence of some factors on primary and secondary outcomes. In their analyses, younger age, low anxiety scores and improvement in fear avoidance beliefs associated to work were predictors of return to work in the group receiving additional work-focused intervention. Authors concluded that the addition of a work-focused intervention may be a better option than standard multidisciplinary intervention for some patients.

Multidisciplinary Interventions

Multidisciplinary interventions are characterized by teams including several professionals with different backgrounds, who evaluate and intervene in different areas involved in the participation in working life.

Studies Reporting Positive Change

Poulsen and colleagues [21] evaluated in Denmark a multidisciplinary, coordinated and tailored return to work intervention in a RCT with 12 months follow-up and including 3 municipalities. Municipalities are obliged by law to conduct an assessment of every sick-listed beneficiary by the end of the 8th week of sickness absence. At this assessment, beneficiaries are assigned to one of three categories: (1) likely to return to work within three months; (2) unlikely to return to work within three months, but able to participate in return to work activities like gradually returning to work; and (3) unlikely to return to work within three months and unable to participate in return to work activities. Participants of the study were employed and unemployment adults with chronic disease and disability in general assigned to category 2. Persons receiving the multidisciplinary intervention in the three municipalities (*n* = 747, *n* = 809, *n* = 392) were compared to persons receiving ordinary sickness benefit management (*n* = 489, *n* = 539, *n* = 129). The effect of the multidisciplinary strategy was different in the 3 municipalities and across time frames within each site. In the municipality with the most complex cases, the intervention was effective regarding recovery from sickness absence, defined as the first week where no sickness absence benefit was given. It is important to stress that the Danish return to work program was performed during a period of global economic crisis (data collection 2010 to 2011) with increasing rates of unemployment throughout Europe. The authors concluded that contextual factors, such as sociodemographic characteristics of participants in the different municipalities and different interpretation and management of legislation, were likely to explain the results.

Jensen and colleagues [28] evaluated in Denmark the same multidisciplinary, coordinated and tailored intervention for employed persons with low back pain and on sick leave for 3 to 16 weeks. The multidisciplinary intervention consisted of: clinical examination and advice by a rehabilitation doctor and a physiotherapist; assignment of a case manager, who developed a rehabilitation plan in collaboration with the patient and a multidisciplinary team; contacting the workplace and the social service center to discuss and coordinate relevant initiatives; arranging meetings between the participant and each of the other specialists, meetings at the work place and meetings with the social service centre, if relevant. In a RCT with 12 months follow-up, 176 persons (54% females) receiving multidisciplinary tailored coordinated intervention were compared to 175 persons (50% females) receiving a brief intervention consisting of clinical examination and advice. There were no differences between groups in number of subjects who returned to work within one year or time needed to return to work. Return to work was defined as the first 4-week period within the first year after inclusion, during which the participant received no social transfer payments. Stapelfeldt and colleagues [30] carried out a secondary analyses of this study to identify subgroups that would benefit more from the multidisciplinary intervention, considering the 12 months follow-up and using data from 120 persons. When claimants, i.e., persons applying for pension, were excluded from the analyses, the multidisciplinary intervention was more effective for participants with low job satisfaction and in subgroups characterized by no influence on work planning and at risk of losing their job. Participants with high job satisfaction and those who were able to influence the planning of their work and had no risk of losing their job benefited more from the brief intervention. Using a longer follow-up of 24 months, Jensen [29] analysed in a subsequent study the impact of the interventions on sick leave weeks and on different subgroups. In the general sample, at the one-year follow-up the number of weeks on sick leave was statistically significant lower in the brief intervention group than in the multidisciplinary group which indicated that this intervention was the more effective one. When authors focused on different subgroups of patients, the brief intervention worked better for about two thirds of the patients, namely those with influence on the planning of their own work and no perceived risk of losing job and/or being a work injury claimant, and the multidisciplinary intervention was more effective for the remaining one-third of the patients.

Vermeulen et al. [31] evaluated in the Netherlands a multidisciplinary intervention promoting involvement of stakeholders (*n* = 84, 43% females) compared to usual care (*n* = 79, 37% females) in a RCT with 12 months follow up. Participants were persons with musculoskeletal disorders, unemployed and temporary registered on sick leave for 2 to 8 weeks. In the intervention group, a return to work coordinator encouraged a high degree of involvement of both the sick-listed worker and labour experts representing the Social Security Agency to reach consensus about a return to work plan. A vocational rehabilitation agency was contracted to find a suitable (therapeutic) workplace matching with the formulated return to work plan. Results indicated a non-significant trend towards delayed return to work in persons receiving the intervention in the first 90 days, followed by a significant advantage in sustainable return to work rate after 90 days. Sustainable return to work was defined as days from randomization to work in any type of paid work or work resumption with ongoing benefits for at least 28 consecutive days.

Studies Reporting No Change

Johansson and colleagues [20] evaluated the program Resursteam, a multidisciplinary collaboration program in Sweden focusing on an early and holistic evaluation of the needs for rehabilitation as a collaboration between the Social Insurance Agency and the primary health care. Goal of Resursteam was to speed up rehabilitation and to reduce work absence costs. Resursteam was evaluated for persons with chronic diseases and disability in general in a mixed methods study including a one year follow up RCT and a register-based cohort study with an approximately 3 years follow up. Participants were employed and unemployed persons on sick leave and at risk of becoming long-term sick. In the cohort study and in the RCT, 1076 and 21 persons received Resursteam, respectively. In the comparison group, 37,938 persons in the cohort study and 24 in the RCT received the rehabilitation plan suggested by the medical doctor and/or the case worker. The results from the cohort study suggested a negative effect of the intervention: the duration of sickness absence of persons receiving Resursteam was about 3 months longer. Despite controlling for possible confounders, groups were initially very different in the cohort study because Resursteam is prescribed to people with risk of long sick absence. However, results from the small RCT, albeit not significant, were consistent with results of the cohort study.

Steiner and colleagues [27] evaluated a multidisciplinary functional rehabilitation program (MFRP) in Switzerland including significant cognitive behavioural components and work-related goals and outcomes (integrating physical rehabilitation, psychological evaluation, cognitive behavioural methods and occupational therapy with a socio-professional component) aimed to restore the individual’s musculoskeletal function. The intervention was evaluated in a controlled trial with a 9 months follow up including persons with non-specific low back pain: 24 persons (42% females) received MFRP and were compared to 21 persons (52% females) receiving a muscle reconditioning program. After excluding subjects not employed or not searching for a job, for instance housewives or persons in early retirements, more people who received MFRP were working at the follow-up (78% vs. 47%) but this difference was statistically not significant. It is important to stress that this study had a very small sample size and did not define what was considered return to work.

Educational Strategies

Educational interventions focus usually on information and advice, education about nature and course of the disease and about physical and psychological factors involved.

Studies Reporting Positive Change

The single identified study evaluating an educational strategy was carried out in Belgium by Du Bois et al. [32]. The effectiveness of a disability evaluation followed by information and advice was evaluated in a RCT with 12 months follow-up for employed persons with low back pain on sick leave. The disability evaluation was followed by information and advice including education about nature and course of the disease and about physical and psychological factors involved as well as encouraging participants to adopt an active role. Persons receiving the disability evaluation followed by information and advice (*n* = 252, 46% females) were compared to persons receiving usual care including the brief disability evaluation but no medical advice (*n* = 257, 40% females). The educational intervention was more effective in the long term: less people receiving disability evaluation followed by information and advice were off work or had episodes of sick leave after 12 months. Time until recurrent sick leave was also lower for this group.

Studies Reporting No Change

No study identified.

## 4. Discussion

With this systematic review—carried out in the scope of the EU-funded project PATHWAYS—we evaluated the effectiveness of strategies for integration and re-integration to work for persons with chronic diseases and disability in general, and for persons with musculoskeletal disorders, implemented in European countries. A total of 21 publications of reliable methodological quality and published in English between January 2011 and April 2016 were included. A quantitative summary of the effect of reported interventions was not considered appropriate because of the methodological differences between studies. Considering persons with chronic diseases and disability in general, we observed positive changes in employment status, return to work and sick leave outcomes for graded sickness-absence certificates in Norway, for PTSL in Denmark and Finland, and for a multidisciplinary, coordinated and tailored return to work intervention in Denmark. Considering persons with musculoskeletal disorders, we observed positive change in the same work outcomes for PTSL in Sweden, for the multidisciplinary, coordinated and tailored return to work intervention in Denmark, for a multidisciplinary intervention promoting involvement of stakeholders in the Netherlands and for disability evaluation followed by information and advice in Belgium. Additionally, the co-existence of (a) active labour market policies to promote the employment of persons with disability and (b) passive measures to support persons with disability (e.g., disability pensions) has been explored in Italy and a positive association between the co-existence of both measures and the probability of finding a job for individuals with a disability was found.

One might argue why we included publications focusing on persons with disability in a systematic review evaluating strategies for persons with chronic conditions. The answer to this question is straightforward: because persons with chronic conditions experience considerable disability in daily life, ranging from problems in body functions such as problems with pain, the level of energy or muscle power, to limitations in activities such as doing housework or walking, and important restrictions in their participation in society, such as keeping a job and raising a family. This fact has been corroborated by diverse studies [2,39] and is also reinforced by the Global Burden of Disease Study, which overwhelmingly identified NCDs, many times chronic conditions, as the ones mostly associated to disability [40]. A recent article on disability, NCDs and health information stresses that it is very unfortunate that action plans for NCDs, and their corresponding monitoring frameworks, continue to heavily focus on mortality and to neglect the adequate measurement of both morbidity and disability [41]. On the same token, it is unfortunate if policies for persons with disabilities rarely reach people with chronic diseases, as recently shown in an overview of European measures to manage and support work participation [11]. In fact, the majority of people who receive disability benefits in Europe have chronic diseases [13]. The fact that legislation for persons with disabilities does not always benefit people with chronic diseases might be a matter of the way how disability is defined in the country. Countries using a narrow definition of disability as a personal characteristic of a minority, such as blindness or deafness, considerably restrict the reach of their laws, while countries adopting a more inclusive definition, in line with the definition proposed by WHO in the International Classification of Functioning, Disability and Health (ICF) [42] will considerably broaden the population that benefits from disability policies. As recently stated, it is time to rethink disability [43]. This is true also in the scope of strategies for integration and re-integration to work of persons with chronic diseases.

A core finding of this systematic review is the urgent need to improve the methodology of evaluations targeting effectiveness of strategies aiming integration and re-integration into work for persons with chronic diseases. The present work unveiled several barriers hindering the comparability of scientific studies, such as a large variability on how the core work outcome of interest, for example return to work, is defined and measured. Out of 32 papers initially identified, 11 were not included in the present review because their shortcomings in study design and in reporting were considered likely or very likely to change study’s conclusions regarding the work outcomes of interest. While the number of identified studies shows that research is done in the area of integration and re-integration into work, methodological shortcomings stress the need to improve their quality considerably. For instance, a standard inclusion of (a) comparison group(s), a better description of characteristics of non-randomized groups as well as avoiding high percentages of persons lost to follow up (or evaluating the reasons behind it when it happens) would be important. We recommend that evaluations alongside implementations of policies, systems and services are planned in detail using structured research protocols. Additionally, the inclusion of control groups or measures to guarantee comparability with usual “treatment”, alternative interventions or “natural” trajectories of work problems should be mandatory, so that results can be indeed attributed to the interventions. Our call for better research is in line with recent publications highlighting similar issues and calling for sounder methodologies to evaluate strategies targeting integration and re-integration into work. For instance, a recent systematic review investigating the effectiveness of workplace disability management programs that promote return to work reported that insufficient data on sample characteristics were available and that effect sizes were uncertain [44]. Authors concluded that the evidence needed to confirm the effectiveness of workplace disability management programs is insufficient. Similarly, a more solid integration of research on return to work in healthcare centers or outpatient clinics and cluster randomization have been recommended in another systematic review to foster the inclusion of under-represented groups, such as men or less-educated individuals [45].

Most effectiveness evaluations have been conducted in Nordic countries and most strategies leading to positive results were as well implemented in those countries. Interventions targeting integration and re-integration into work are strongly intertwined in the welfare models of the country where they are implemented. The Scandinavian social welfare model emphasizes egalitarianism and universal welfare provision [46] and generally provides universally accessible benefits and a strong redistributive social security system [47,48]. In the present review, graded sickness-absence certificates, PTSL, and multidisciplinary, coordinated and tailored return to work interventions were effective in improving the employment status, return to work and sick leave outcomes of persons with chronic conditions living in Nordic countries. Although these interventions have been successful in countries where formal regulations encouraging employees, employers, and physicians to use the system are in place, they might be successfully implemented in other European countries. For instance, a multidisciplinary intervention promoting involvement of stakeholders was also effective in the Netherlands [31]. However, it is important to stress that an adaptation of these strategies to other welfare models and the consideration of laws and regulations in the country are essential to increase their success likelihood. An overview of European welfare models and the state of the art of strategies for professional integration and reintegration of persons with chronic diseases is reported in detail in another publication of this special issue [11].

It seems worth to broaden the evaluation of the effectiveness of combining passive and active strategies for integration and re-integration into work for persons with chronic conditions. This review points out through the work of Agovino and Rapposelli [14] in Italy that combining active strategies, such as supported employment or active labour market policies, with passive strategies, such as disability benefits, is a promising way of effectively keeping persons with chronic conditions at work. This is strengthened by the work of Lopez Frutos [19] in Spain showing that receiving disability benefits didn’t decrease the probability of working for individuals with moderate levels of disability, as usually feared. How passive and active strategies can be best combined, and how much of each bring the best results are, however, still open questions. Such combinations, also called Flexicurity, are a core topic of current European debates about social security reforms, having being integrated in the European Employment Strategy [49]. Sound research in this area is therefore highly recommended. It is important to stress though that research on the effectiveness of policy strategies is complex, requires good quality and accessible register data and sophisticated data analyses approaches. It is therefore essential that Governments foster policy research by investing resources in large and reliable register databases, as recommended by the PATHWAYS project [50].

Multidisciplinary interventions are promising strategies that might meet the complexity of needs of persons with chronic health conditions. A further study of the PATHWAYS project on the needs of persons with chronic conditions [51] has shown that return or maintenance of work is a complex process and that the needs associated to work life are multifold. Participants of this survey named a variety of work-related aspects—among others career development, stress at the workplace, work structure and schedule, and workload—support and attitudes of colleagues and co-workers, health-related and person-related aspects as factors that impact their work lives. Multidisciplinary interventions have the potential to meet this complexity. They are characterized by teams including several professionals with different backgrounds, who evaluate and intervene in different areas involved in the participation in working life. In the present review, four out of the seven publications evaluating multidisciplinary interventions reported positive results. In the case of negative or inconclusive results, secondary analyses might help disclosure the reasons behind it. For instance, Jensen and colleagues [28] evaluated a multidisciplinary, coordinated and tailored intervention for employed persons with low back pain and on sick leave but could not prove the intervention was effective. Stapelfeldt and colleagues [30] carried out a secondary analysis of this study and unveiled that when persons applying for pension were excluded from the analyses, the multidisciplinary intervention was more effective for participants with low job satisfaction and in subgroups characterized by no influence on work planning and at risk of losing their job. Using a longer follow-up of 24 months, Jensen analyzed in a further secondary analyses [29] the impact of the intervention on sick leave weeks. At the one-year follow-up the number of weeks on sick leave was statistically significant lower in the control group than in the multidisciplinary group. However, when authors focused on different subgroups of patients, the brief intervention worked better for patients with influence on the planning of their own work and no perceived risk of losing job and/or being a work injury claimant, while the multidisciplinary intervention was more effective for the remaining patients. What this example clearly shows is that there is not a standard solution for all persons with chronic conditions and that it is important to define target groups in more detail and design interventions suitable for them. This example also points out to the importance of one of the policy recommendations of the PATWAYS project [50]: the need for individualized tailored strategies.

The availability of part time sick leave may be effective in promoting return to work in many cases. We shouldn’t forget, however, that PTSL worked well where the social system compensates the employee for the loss of wage due to the reduced working hours. The employer may still have to re-organize work, which might cost time and financial resources, but is free from further financial burden. The existence of additional measures for employers, such as financial aids for accommodations or expert support in the communication between employer and employee, might further facilitate the process of return to work. Our positive findings regarding PTSL are supported by available literature [52,53,54]. The question on whether part time sick leave is associated with sustainable and successful return to work must be further investigated though, as employment status at a specific and fixed time points provides relatively limited information about the effect of a strategy in the middle and long run. Additionally, since the specialty of medical doctors certificating the sick leave seems to affect the probability of being in part or full time sick leave, this should be further investigated as well [38]. Finally, it is important to stress that some studies show no effect of sick-leave. For instance, a Norwegian cluster-randomized study on ‘‘active sick leave’’, which implies returning to an adjusted work environment with the assistance of social security, showed no beneficial effects [55]. These studies point out potential weaknesses that need to be better understood.

A main goal of the EC-funded PATHWAYS project is to provide stakeholders with recommendations on which interventions are available, effective and could therefore potentially be transferred from one European country to others. This systematic review identified many effective interventions. However, it is important to stress that several factors, such as the welfare model of countries, the laws in place regulating employment and the cooperation between different agencies involved in return to work strategies play an important role and must be considered. Lopez Frutos [19] stressed that factors, such as discrimination, the lack of jobs that are adequate for the limitations of the person or the structure of the disability system, can considerable affect return to work. Furthermore, they point out that changes in policy must be carefully planned, including the consideration of potential welfare gains and losses of all agents involved. For instance, if there are no compensations (e.g., wage compensation, taxes reductions) for companies employing persons with higher levels of disability, the employment inequalities faced by this group may be perpetuated, particularly in countries with high unemployment rates. Indeed, a further PATHWAYS publication [50] showed that both national and European stakeholders are very critical regarding legislative frameworks and the coordination necessary for a sustainable implementation of employment re-integration policies, and considered existing policies and coordination strategies in Europe as inadequate and ineffective, respectively. The same study concludes that the dissemination and broader implementation of effective strategies must be accompanied by appropriate policies fostering a constructive cooperation among key stakeholders, by measures to raise awareness about persons with chronic conditions and work among employers, and by sound monitoring systems.

This review must be appraised in the light of its limitations. A limitation of the present review is that it included scientific publications published between 2011 and 2016. This short period of time of five years was selected because the aim of the PATHWAYS project was to provide an overview of the amount of research along different diseases, countries and of the kind of strategies evaluated. It was outside the scope of the pathways project to perform an exhaustive review of specific interventions, as done for instance by Gensby et al. in 2013 [44]. A second limitation is that almost all evaluations were therefore carried out either during or right after the financial crisis of 2008, especially when literature shows that return to work is more difficult during or right after economic recessions [56]. However, positive results were observed in our review. Further limitations are that we searched for literature only in English, although the publications of interest might be frequently published in the languages of the countries implementing them, that we could not provide a quantitative synthesis due to the heterogeneity of employment outcomes focused in the included studies, that our inclusion criteria regarding the study population focused either on musculoskeletal diseases in general or on back or neck pain, excluding further musculoskeletal disorders, such as arthrosis or osteoporosis, that we restricted the review to European studies, although a comparison with international evaluations could have provided insights on why some interventions were effective and others not, and that studies generally exclude persons working independently, such as architects or lawyers. Due to the scope of this work, we focused on employment outcomes and left out potentially important outcomes, such as health outcomes and quality of life, on which interventions might have had an impact. Finally, we could have found papers, especially papers evaluating policy strategies, if databases specialized in law, policy or economy would have been included. However, this was beyond the scope of the PATHWAYS project and therefore not done.

One strength of this review is that although it focuses on effectiveness of interventions, observational studies have been included. This was an important decision; otherwise we would have missed data on some measures, such as PTSL, that are difficult to evaluate because of their characteristics. Non-randomized intervention studies and well-defined observational cohort studies may provide this data—particularly if analyses are adjusted for potential influencing variables and investigate different scenarios through sensitivity analyses. Consequently, knowledge, although of a lower degree of confidence, may accumulate. However, we still must be particularly careful when considering the results of non-randomized studies, particularly observational ones, and be aware that conducting studies with a more adequate design to assess effectiveness is complex but possible, if management levels are sensitized, as shown in two exemplary studies [21,37].

*A final open question is why several of the interventions targeted to persons with chronic diseases in general were not effective.* One possible explanation for the lack of conclusive results might be the heterogeneity of the populations included in terms of chronic conditions, disease severity, prognosis as well as treatment and work modifications options. Indeed, in a recent systematic review assessing the effects of various measures targeted at enhancing return to work, there were no or mixed effects in populations with non-specified sick absence in terms of disease and severity [57]. If possible, secondary analyses focusing on more homogeneous groups should be carried out, especially when studies fail to confirm effectiveness. Another reason could be that the intervention was not implemented as intended (e.g., it didn’t reach the target population, the skills of professionals involved didn’t fit the abilities needed in the praxis, different definition of goals and methods by different institutions involved in the process of work integration). Qualitative studies and process evaluations are needed shed light on hindering and facilitating factors that influence effectiveness.

## 5. Conclusions

This systematic review showed that positive changes in employment status, return to work and sick leave outcomes of persons with chronic diseases and with disability in general can be facilitated with graded sickness-absence certificates, PTSL, early ergonomic interventions for back pain, disability evaluation followed by information and advice, and with multidisciplinary, coordinated and tailored return to work interventions. Additionally, the review found a positive association between the co-existence of (a) active labour market policies to promote employment and (b) passive support measures (e.g., pensions) and the probability of finding a job. However, a core finding of this review is the urgent need to improve and strengthen research on the evaluation of the effectiveness of strategies targeting integration and re-integration into work for persons with chronic health conditions.

## Figures and Tables

**Table 1:** Characteristics of included publications by disease and kind of strategy. Abbreviations: CG: comparison group; IG: intervention group; FU: follow-up; n.a.: not applicable; n.r.: not reported; PwD: persons with disability; SL: sick leave; MSD: musculoskeletal disorders; RTW: return to work; CBT: cognitive-behavioral therapy; PTSL: part-time sick leave; FTSL: full-time sick leave; LBP: low-back pain.

First Author Year Country Reference	Strategy Study Design Subjects Follow up	Effect on Work-Related Outcomes Supported by Data (Yes/No/Unclear) Employment-Related Outcomes	Further Information
**(A) Chronic Disease or Disability**			
**Policy Strategies**			
Agovino M. 2015 Italy [14]	**Strategy: co-existence of active labour market policies and passive measures to support PwD** Design: cross-sectional, register-based Subjects: people with a disability CG: n.a. IG: n.a. FU: n.a.	**YES** The combination of active labour market policies to promote the employment of PwD and passive measures to support PwD (i.e., disability pensions) was positively related to the probability of finding a job (*p* < 0.05) after controlling for labour market variables. RELEVANT OUTCOMES (1) percentage of PwD that are employed	Women: n.r. Yearly data for the period 2006–2011. Strategy: co-existence of (a) active labor market policies to promote the employment of PwD and (b) passive measures to support PwD. The combination of active and passive measures is at the core of the concept of “flexicurity”, a strategy to promote, both flexibility and security in the labour market. The authors calculated three flexicurity indexes that give different weight to passive and active measures and explored their correlation with the probability of finding a job for PwD in the different Italian regions.
**Disability Benefit**			
Lopez Frutos E.M. 2015 Spain [19]	**Strategy: disability support benefit** Design: cross-sectional, register-based Subjects: PwD CG: Certificate of disability without disability support benefit; *n* = 19,976 IG: Certificate of disability and disability support benefit; *n* = 27,660 FU: n.a.	**NO** Being entitled to a disability support benefit showed a significant negative association with the probability of working for individuals in the disability threshold (disability level of 33–44%) after controlling for health and sociodemographic variables (19.3% lower probability of working). For individuals with a degree of disability ≥ 45% there was no statistically significant difference in the probability of working for those receiving a benefit. RELEVANT OUTCOMES (1) employment status	Women 45.6%. The sample includes all individuals that held a certificate of disability in 2008, 2009 or 2010. The certificate of disability is an administrative acknowledgement of a disability degree of 33% or more. Persons with the disability certificate have financial and tax advantages. In addition, persons entitled to a disability support benefit receive a monthly allowance. They are also required to find a different job to the position they had before the disability.
**Multidisciplinary Intervention**			
Johansson P. 2012 Sweden [20]	**Strategy: early and holistic evaluation of the need for rehabilitation** Design: mixed methods (a-RCT; b-cohort study, register-based) Subjects: individuals on SL and at risk of becoming long-term sick, employed and unemployed CG: usual care; cohort study, *n* = 37,938; RCT, *n* = 24 IG: Early and holistic evaluation of the need for rehabilitation; cohort study, *n* = 1076; RCT, *n* = 21 FU: RCT approx. 1 year; cohort study approx. 3 year	**NO** The results from the RCT and the retrospective observational study (controlling for health and sociodemographic variables) did not support a positive effect of the intervention: the sick-spells of the IG lasted longer. RELEVANT OUTCOMES (1) duration of sickness absence Duration of sickness absence: time until leaving the sick spell.	Women: RCT 71–76%; Cohort study 61–65%. RCT: year 2006; Cohort study: 2004–2007; within-subjects analyses 2001–2003 and 2004–2007. Intervention: Multidisciplinary collaboration program (“Resursteam”). Collaboration between the Social Insurance Agency and the primary health care. The sick-listed individual’s medical doctor, her/his case worker, a behaviorist, and a physiotherapist meet regularly to discuss and assess the insured individual’s need for rehabilitation. Goal: to speed up the rehabilitation and reduce absence costs. Comparator: the medical doctor and/or the case worker should suggest a rehabilitation plan.
Poulsen O.M. 2014 Denmark [21]	**Strategy: multidisciplinary, coordinated and tailored RTW intervention** Design: RCT in 3 municipalities: M1, M2, M3 Subjects: adults receiving long-term (≥8 weeks) benefits, employed and unemployment, unlikely to RTW within three months CG: standard management; *n*: M1 = 489; M2 = 539; M3 = 129 IG: Multidisciplinary, coordinated and tailored RTW; *n*: M1 = 747; M2 = 809; M3 = 392 FU: 12 months	**YES** The effect was different in the 3 municipalities and across time frames within each site. In the municipality with the most complex cases, people in the intervention group showed an increased rate of recovery from long-term sickness absence (HR 1.51, 95% CI 1.31–1.74). RELEVANT OUTCOMES (1) recovery from sickness absence Recovery from sickness absence: first week where no sickness absence benefit was given.	Women: 49.5–62.8%. Data collection: 2010–2011. The municipalities are obliged by law to conduct an assessment of every sick-listed beneficiary by the end of the 8th week of sickness absence. At this assessment, beneficiaries are assigned to one of three categories: category l = likely to return to work within three months; category 2 = unlikely to return to work within three months, but able to participate in RTW activities like gradually returning to work; and category 3 = unlikely to return to work within three months and unable to participate in RTW activities. All category 2 beneficiaries were included in the trial. CG: ordinary sickness benefit management. IG: Intervention includes designated RTW coordinators and multidisciplinary teams. Work accommodation by health providers was used when appropriate.
**Part-Time Sick Leave (PTSL)/Part-Time Sick Benefits**			
Høgelund J. 2012 Denmark [22]	**Strategy: part-time sick leave** Design: cohort, survey and register-based Subjects: people with health problems, employed and on SL >8 weeks CG: FTSL; *n* = n.r. IG: PTSL; *n* = n.r. Total sample: 226 with mental health issues and 638 with other disorders FU: up to 79 weeks	**YES** PTSL significantly reduced the duration of SL for employees with health problems other than mental health issues. RELEVANT OUTCOMES (1) time until first return to regular working hours (RWH) RWH: time until the sick benefit ends because the employee report being ready for return to pre-sick leave hours (examples of reasons to end the sick benefit not considered RWH: receipt of disability benefit, flexijob employment, vocational rehabilitation, end of the normal one-year sickness benefit).	Women: employees with non-mental disorders 61% in PTSL and 55% in FTSL. The benefit cases were closed from 1 January through 31 July 2006. These individuals were interviewed by telephone from March through May 2007, on average ten months after their benefit case ended (and the payment of sickness benefit ceased) and 19 months after the sick leave spell started.
Markussen S. 2012 Norway [23]	**Strategy: graded sickness-absence certificate** Design: cohort, register-based Subjects: people on SL > 8 weeks CG: non-graded absence certificate; *n* = 261,596 IG: graded sickness-absence certificate before the end of week 8; *n* = 77,655 FU: 2 years	**YES** Persons with a graded long-term absence certificate showed significant shorter absence durations, less subsequent social security dependency, and higher employment rates (e.g., the expected number of work-days was reduced more than 90 days, the number of saved social security days was around 80–90 days, and the employment probability two years after the sick spell was about 16-fold higher compared to persons receiving a full-time absence certificate). RELEVANT OUTCOMES (1) number of days from the start to the stop of the absence spell (including holidays and days off), (2) number of lost full-time equivalent working days, (3) number of full equivalent days in social security during the 24 months following the end of the spell (4) employment in the 2nd year after starting the spell	Women: CG 53.0%; IG 67.8%. Data collection: 2001–2006.
Kausto J. 2012 Finland [24]	**Strategy: partial sick leave** Design: cohort, register-based Subjects: people with MHP, MSD, cancer and trauma; employed and on SL at least for 60 days, working full time before their leave period CG: FTSL; *n* = 28,380 IG: PTSL; *n* = 1047 FU: approx. 12–19 months	**YES** PTSL was associated with increased subsequent use of partial disability pension (8%, 95% CI 10% to 5%) and decreased use of full disability pension (6%, 95% CI 3% to 9%). The effect was stronger for men (5% and 10%, respectively). Overall results suggest enhanced work retention after PTSL. RELEVANT OUTCOMES (1) maintaining work Full disability pension as an indicator of leaving of the labour market and partial disability pension as indicator of retaining the job despite impaired work ability.	Women Analysis performed with all subjects: CG: 53%, IG: 72%. Analysis performed with matched sample CG: 72%, IG: 72%. Recipients of partial or full sickness benefit whose sick leave period had ended between 1 May and 31 December 2007 were included.
Kausto J. 2014 Finland [25]	**Strategy: partial sickness leave** Design: cohort, register-based Subjects: people with musculoskeletal diseases, mental disorders, traumas and tumours; employed and on SL at least for 60 days CG: FTSL; *n* = 56,574 (matched subsample, *n* = 1660) IG: PTSL; *n* = 1738 (matched subsample, *n* = 1660) FU: 12 months	**YES** Work participation in the IG decreased less than in the CG (difference = 5.3%, 95% CI 3.1% to 7.5%). A larger effect was seen in people aged 45–65 years. In analyses with matched subsamples the effect on work participation was stronger (difference = 9.8, 95% CI 5.9% to 13.7%) and shown in all age groups (16–65 years). RELEVANT OUTCOMES (1) work participation Work participation: time the individuals were likely to have participated in gainful employment; approximated as the proportion of time within 365 days when participants had an employment contract and did not receive either partial or full ill-health-related benefits or unemployment benefits.	Women Analysis performed with all subjects: CG: 53%, IG: 71%. Individuals who had received either partial sickness benefit or full sickness benefit in 2007–2008 and whose compensated sickness absence period had ended between 1 January and 31 December 2008 were included. Analyses for the whole population were adjusted for age, sex, income, diagnosis, occupational group, insurance district. Further analyses were performed for matched subsamples similar in age, gross income, number of unemployment days, sickness absence days, rehabilitation days or work participation before the intervention.
**Notification of Sickness Absence**			
Halonen J. 2016 Finland [26]	**Strategy: legislative changes obligating notification of prolonged sickness absence and assessment of remaining work (“30-60-90 day rule”)** Design: cohort, register-based Subjects: public-sector employees with permanent job contract and on SL for at least 30 calendar days Cohort 1 (reference) *n* = 6393 Cohort 2 (pre-intervention) *n* = 6011 Cohort 3 (intervention) *n* = 5708 FU: 12 months	**YES** Workers who had been 60 days on sick leave returned to work earlier after introduction of the “30-60-90 day rule” (*p* = 0.017). The gain in work participation was larger for women than for men (287.8 vs. 70.4 persons-years/10,000 employees) and for the low than the high job status group (409.7 vs. −30.4). The effects diluted over time. RELEVANT OUTCOMES (1) sustainable RTW after 30, 60 and 90 SL-days, (2) monthly work participation after 30, 60 and 90 SL-days (3) gain in annual work participation after 30, 60 and 90 SL-days Sustainable RTW: a minimum of 28 consecutive working days after the sick absence.	Women: approximately 75% (most participants were women due to the nature of public sector jobs in Finland). Three cohorts: 2008/9 (reference), 2010/11 (pre-intervention), 2013/14 (post-intervention). Covariates: sex, age and occupational status. The total sickness absence rates declined from 2008 until 2013 in both the public and the private sector. The gains in work participation days were larger during the intervention than the reference period, suggesting a beneficial effect of the legislative changes.
**(B) Musculoskeletal Disorders**			
**Multidisciplinary Interventions**			
Steiner A.S. 2013 Switzerland [27]	**Strategy: multidisciplinary functional rehabilitation program** Design: controlled trial Subjects: non-specific LBP CG: muscle reconditioning program (MRP); *n* = 21 IG: Multidisciplinary functional rehabilitation program (MFRP); *n* = 24 FU: 9 months	**UNCLEAR** After excluding subjects not employed or not searching for a job (e.g., housewives or early retirements), more people in the IG were working at follow-up (78% vs. 47%) but the difference was not significant. RELEVANT OUTCOMES (1) RTW (not further described)	Women: CG 52%, IG 42%. Data collection: CG mid-2006-mid 2007, IG end of 2007 to 2008 Intervention: It integrated physical rehabilitation, psychological evaluation, cognitive behavioural methods and occupational therapy with a socio-professional component. Participants in the CG received MRP, the former standard treatment at the study clinic. After the center replaced MRP by MFRP, the IG received the new standard treatment.
Jensen C. 2011 Denmark [28]	**Strategy: multidisciplinary tailored coordinated intervention** Design: RCT Subjects: LBP, employed and on SL for 3–16 weeks IG1: Brief intervention (clinical examination and advise); *n* = 175 IG2: Multidisciplinary tailored coordinated intervention; *n* = 176 FU: 12 months	**NO** There were no differences in number of subjects who achieved RTW (76.0% in IG1 and 71.0% in IG2) and time to RTW (14 weeks in IG1 and 18 weeks in IG2). RELEVANT OUTCOMES (1) RTW (2) Median time until RTW RTW: first 4-week period within the first year after inclusion without social transfer payments; unemployed participants were classified as “RTW,” if they had lost their job during follow-up, but were healthy enough to work, which was a prerequisite to receive unemployment benefits.	Women: CG 50%, IG 54%. Recruitment: November 2004–June 2007. Multidisciplinary Intervention: Clinical examination and advice by a rehabilitation doctor and a physiotherapist; assignment of a case manager, who develops a rehabilitation plan in collaboration with the patient and a multidisciplinary team; the workplace and the social service center are contacted to discuss and coordinate relevant initiatives; the case manager arranges meetings between the participant and each of the other specialists, meetings at the work place and meetings with the social service centre, if relevant. Sample: Specific and non-specific LBP; 56% unskilled worker; >80% wished to get back to same work.
Jensen C. 2012 Denmark [29] (same study as Jensen C. 2011)	**Strategy: multidisciplinary tailored coordinated intervention** Impact of the interventions on sick leave weeks and on different subgroups explored; longer FU than Jensen 2011 FU: 24 months	**YES—SUBGROUP DIFFERENCES** Results for the general sample: at the two-year follow-up, no statistically significant difference between the brief intervention group and the multidisciplinary group was found. Results for subgroups of patients: - The brief intervention seemed to work better for about two thirds of the patients, (with influence on the planning of their own work and no perceived risk of losing job and/or being a work injury claimant (82% of subjects in IG1 returned to work within 2 years vs. 75% in IG2; *p* = 0.028); - multidisciplinary intervention was more effective for the remaining one-third of the patients (65% of subjects in IG2 had returned to work at the two-year follow up vs. 51% in IG2; *p* = 0.098). The other outcome measures showed the same tendency, but the differences were not statistically significantly different. RELEVANT OUTCOMES (1) time to RTW at 1 and 2 years (2) RTW during follow up (3) work status at 1 and 2 years (4) SL weeks (partial or full) at 1 and 2 years RTW: 4-week period without sick or other health-related benefitsOnly sick leave spells of ≥2 weeks were considered	
Stapelfeldt C.M. 2011 Denmark [30] (same study as Jensen C. 2011)	**Strategy: multidisciplinary tailored coordinated intervention** Secondary analyses to identify subgroups that would benefit more from the multidisciplinary intervention; FU considered: 12 months. It also analyses data from further 120 subjects (IG1 *n* = 60; IG2 *n* = 60)	**YES—SUBGROUP DIFFERENCES** When claimants were excluded from the analyses, the multidisciplinary intervention was more effective in the subgroup of participants with low job satisfaction and in subgroups characterised by no influence on work planning and groups at risk of losing their job. Participants with high job satisfaction and those who were able to influence the planning of their work and who had no risk of losing their job benefited more from the brief intervention. RELEVANT OUTCOMES (1) RTWRTW: no sick leave compensation for a period of 4 consecutive weeks.	
Vermeulen S. 2011 The Netherlands [31]	**Strategy: multidisciplinary intervention promoting involvement of stakeholders** Design: RCT Subjects: MSD, unemployed and temporary agency workers on SL 2 to 8 weeks CG: usual care; *n* = 84 IG: multidisciplinary intervention; *n* = 79 FU: 12 months	**YES** The results indicated a non-significant trend towards delayed RTW in the IG in the first 90 days, followed by a significant advantage in RTW rate after 90 days (HR 2.24; (95% CI 1.28–3.94). The intervention had a negative impact on sickness benefit duration, although not statistically significant. This was due to the fact that in most cases the therapeutic workplaces were offered with ongoing sickness benefit. RELEVANT OUTCOMES (1) time to sustainable first RTW at 3, 6, 9 and 12 months (2) time to first sustainable ending of sickness benefit (3) total number of days of sickness benefit at 3, 6, 9 and 12 months Sustainable first RTW: days from randomisation to work in any type of paid work or work resumption with ongoing benefits for at least 28 consecutive days. First sustainable ending of sickness benefit: duration in calendar days from the day of randomization until ending of sickness benefit for at least 28 days. Recurrence of sickness absence with an accepted sickness benefit claim within 28 days after ending of the previous sickness benefit was considered as belonging to the preceding sickness benefit period, on condition that it was due to the same (or related) MSD.	Women: CG 37%, IG 43%. Recruitment: March 2007–September 2008. Comparison: assessment and management of vocational rehabilitation carried out by an insurance physician, a labour expert and a case-manager. Intervention: a RTW coordinator work to stimulate a high degree of involvement of both the sick-listed worker and the labour expert (representing the Social Security Agency), and to reach consensus about a RTW plan. A vocational rehabilitation agency was contracted to find a suitable (therapeutic) workplace matching with the formulated RTW plan. Sample: Volunteers (/interested in participation).
**Educational Strategies**			
Du Bois M. 2012 Belgium [32]	**Strategy: Information and advice to stay active by medical advisers during after a disability evaluation** Design: RCT Subjects: LBP, employed and in SL CG: disability evaluation; *n* = 257 IG: disability evaluation followed by information and advice; *n* = 252 FU: 12 months	**YES** This intervention was more effective in the long term. Less people in the IG were off work (4% vs. 8%) or had episodes of SL (15% vs. 23%) after 12 months. Time until recurrent SL was lower in the IG (59 vs. 71 days). RELEVANT OUTCOMES (1) RTW rate at 3 and 12 months (2) episodes of sick leave for LBP at 3 and 12 months (3) sick leave duration (mean number of days off work) (4) time until recurrent sick absence	Women: CG 40%, IG 46%. Recruitment: March 2008–September 2008. Comparison: brief disability evaluation without medical advice. Intervention: disability evaluation followed by information and advice (education about nature and course of the disease and about physical and psychological factors involved; encouragement of participants to adopt an active role).
**Work-Focused Interventions**			
Jensen L.D.2012Denmark[33]	**Strategy: Counselling addressing workplace barriers and physical activity** Design: RCT Subjects: LBP, employed and expressing concerns about the ability to maintain their current job CG: usual care; *n* = 150 IG: counselling addressing workplace barriers and physical activity; *n* = 150 FU: 3 months	**UNCLEAR** The intervention had a significant effect for self-reports of SL due to LBP for more than 8 weeks (RR 11.78; 95% CI 1.56 to 88.96) and for cumulated SL days due to LPB (RR 2.57; 95% CI 1.52 to 4.37) without considering the approx. 25% loss to FU. However, per register data on SL of more than 2 weeks due to all causes (outcomes available for all participants), there was no significant difference between the CG and the IG (with and without considering patients lost to FU). RELEVANT OUTCOMES (1) proportion of patients accumulating 8 weeks of sick leave (2) duration of sick leave	Women (based on individuals who completed baseline and follow up): CG (*n* = 114) 59%, IG (*n* = 110) 51%. Recruitment: November 2006–April 2009. Intervention: counselling by an occupational physician, aiming at removing experienced workplace barriers as well as at enhancing physical activity of moderate intensity, on pain, function and sick leave after 3 months. Two counselling sessions integrated in LBP secondary care and one workplace visit, if necessary to evaluate the work conditions. Comparison: Usual care would typically consist of a brief instruction in exercises, or readmission to a general practitioner for further contact with a physiotherapist or chiropractic treatment.
Myhre K. 2014 Norway [34]	**Strategy: work-focused intervention additional to multidisciplinary intervention** Design: RCT Subjects: neck and back pain, employed, on sick leave between 4 and 12 weeks CG: multidisciplinary intervention (brief or comprehensive); *n* = 202 IG: additional work-focused intervention; *n* = 203 FU: 12 months	**NO ADDED VALUE TO A MULTIDISCIPLINARY INTERVENTION** Adding work-focus in specialist care did not result in better effect of multidisciplinary interventions. The intervention was not significantly more successful in decreasing time to RTW (except for subjects ≥ 41 y). The intervention had no effect on the total number of subjects achieving RTW. But the work-focused intervention was not inferior to interventions that focus on physical activity and pain. RELEVANT OUTCOMES (1) number of days until sustainable RTW (2) RTWS ustainable RTW: first 5-week period after random assignment without sickness benefits, a work assessment allowance pension, or a disability pension. RTW was designated when patients receiving a partial disability pension prior to inclusion returned to their partial disability.	Women: CG 49%, IG 44%. Recruitment: August 2009–August 2011. Intervention: a case worker analyses together with the patient work and RTW difficulties; they develop a RTW schedule and discuss relevant issues for a meeting with the employer; if sick-leave compensation is an issue, the caseworkers contact municipal social services.
Marchand G.H. 2015 [35] (same study as Myhre K. 2014)	Secondary analysis to explore secondary clinical outcomes and the influence of some factors on primary and secondary outcomes.	**SUPPORT FOR DIFFERENTIAL SUBGROUP EFFECTS** Younger age, low anxiety score and improvement in fear avoidance beliefs of work were positive predictors of RTW in IG as well as in CG.	
Shiri R. 2011 Finland [36]	**Strategy: Early ergonomic intervention** Design: RCT Subjects: upper-extremity pain (different diagnoses), employed CG: Standard medical care; *n* = 86 IG: early ergonomic intervention; *n* = 91 FU: 12 months	**UNCLEAR** The results suggested that an early ergonomic intervention reduces sickness absence due to any MSD. During the 4–12-month period, the number of people with sickness absence due to any MSD was lower in the IG when diagnosed by a nurse (1% vs. 8%, *p* = 0.02) and when certified by physician or nurse (20% vs. 32%, *p* = 0.07). The number of days in sick absence due to any MSD diagnosed by a nurse was significantly lower in the IG when diagnosed by a nurse (*p* = 0.02) but not when certified by physician or nurse (*p* = 0.57). RELEVANT OUTCOMES (1) employees with sick absence in first 3 months and in 4–12 months, (2) sickness absence days in first 3 months and in 4–12 months	Women: 87.3%. Study period: February 2006–December 2007 Intervention: After the clinical examination, the physician contacts the employer, and a visit by the occupational physiotherapist is scheduled. The workplace is assessed and possible changes to achieve an ergonomic improvement discussed with the employee and supervisor.
**Part-Time Sick Leave (PTSL)**			
Viikari-Juntura E. 2012 Finland [37]	**Strategy: Part-time sick leave** Design: RCT Subjects: persons with MSD (neck, shoulders, back and extremities), in SL CG: FTSL; *n* = 31 IG: PTSL; *n* = 31 FU: 12 months	**UNCLEAR** Results suggested better work participation outcomes after PTSL compared with FTSL. Workers on PTSL achieved sooner RTW that sustained at least 4 weeks (12 versus 20 days, *p* = 0.10; adjusted HR = 1.84, 95% CI 1.20–2.82). The number of sickness absence days along the 1-year follow up and the number of recurrent sick leaves per person was about 20% lower in the IG (level of significance not reported). Time to first recurrent sick leave was similar in both groups. RELEVANT OUTCOMES (1) time to sustained RTW (2) number of PTSL-days at 6 time points during 12 month follow-up, (3) number of FTSL-days, (4) proportion of potential work time of the sick days, (5) number of recurrent sick spells per person year, (6) time after end of initial sick leave to the first recurrent sick spell Sustained RTW: the worker continued to work without recurrent sick leave ≥2 weeks or ≥4 weeks after the end of part- or full-time sickness absence.	Women: CG 97%, IG 97%. Recruitment: November 2006–December 2009. Partial sickness allowance was introduced in Finland in 2007. Once introduced, the benefit could be used only after uninterrupted full-time sick leave for >60 working days up to 2010. Research funds were used to compensate the employers for part-time sick leave.
Andren D. 2012 Sweden [38]	**Strategy: part-time sick leave** Design: cohort study, register-based Subjects: MSD, employed and in SL CG: FTSL; *n* = 1037 IG: PTSL; *n* = 133 FU: 330 days	**YES** Workers had a 0.25 higher likelihood of full recovery if assigned to PTSL than FTSL. The average treatment effect of PTSL was 25%. RELEVANT OUTCOMES (1) RTW with full recovery of lost work capacity.	Women: 60%. Selection of subjects: February 2001. PTSL: individuals are covered by the sickness insurance with 25, 50, or 75% sick leave.

**Table 2:** Characteristics of interventions evaluated in publications targeting persons with chronic diseases or disabilities in general. Empty cells mean either that the intervention did not focus on the aspect or that it was not described in the publication.

Author Year	Country	Targeted Population	Strategy (Name)	Strategy (Description)	Multidisciplinary	Interagency/Collaboration	Individualized	Early Intervention	Education Participants	Education Others	Self-Management	Workplace Involved	PTSL Allowed	Data Support Effect on Outcomes
Agovino M. 2015 [14]	Italy	Mixed, people with disability	Flexicurity	Combination of labour market flexibility and high levels of social security.										Yes
Lopez Frutos E.M. 2015 [19]	Spain	Mixed, people with a certificate of disability	Disability support benefit	Disability support benefits provide monthly allowances while, at the same time, requiring the individual to find a job different to the position they had before the disability.								No	Yes	No
Halonen J. 2016 [26]	Finland	Public employees with permanent job contract, on sick leave for ≥30 calendar days	Law- mandated notification of prolonged sick leave (“30-60-90 day rule”)	The legislative change emphasize early notification of both the Occupational Health Service (OHS) and the Social Insurance Institution of prolonged sickness absence as well as the collaboration of the employee, the OHS and the employer in the assessment of possibilities to continue working.		Yes	Yes	Yes				Yes	Yes	Yes
Kausto J. 2012 [24]	Finland	Employed and on long-term sick leave	Partial sick leave	Partial sick leave is indicated if part-time work is not supposed to hinder recovery. It cannot exceed 72 days. Use is voluntary and the decision is taken in collaboration by the patient, the employer and the physician.			Yes					Yes	Yes	Yes
Kausto J. 2014 [25]	Finland	Employed and on long-term sick leave	Partial sick leave	Partial sick leave is indicated if part-time work is not supposed to hinder recovery. It cannot exceed 72 days. Use is voluntary and the decision is taken in collaboration by the patient, the employer and the physician.			Yes					Yes	Yes	Yes
Markussen S. 2012 [23]	Norway	Employed and on long term sick leave	Graded sickness absence certificate	Graded sickness absence certificate within the first 8-weeks of sickness absence and for up to 8-weeks.			Yes	Yes				In part	Yes	Yes
Høgelund J. 2012 [22]	Denmark	Employed and on long-term sick leave (> 8 weeks)	Part-time sick leave	Part-time sick leave allows employees on full-time sick leave to work temporarily at reduced working hours. The employer and the employee must make an agreement about the job contents and working hours. Employee receives the normal hourly wage for the hours worked and sickness benefit for the hours off work, and may gradually increase working hours.			Yes					Yes	Yes	Yes
Johansson P. 2012 [20]	Sweden	Employed and unemployed sick-listed individuals at risk of becoming long-term sick	Resursteam	Multidisciplinary collaboration program consisting of an early and holistic evaluation of the need for rehabilitation. Collaboration between the Social insurance Agency and the primary health care.	Yes	Yes	Yes	Yes				No	Yes	No
Poulsen O. 2014 [21]	Denmark	Working-age adults with a disability receiving long-term sickness benefits (>8 weeks)	Multidisciplinary, coordinated and tailored RTW intervention	Intervention includes designated RTW coordinators and multidisciplinary teams. Work accommodation by health providers was used when appropriate	Yes	Yes	Yes					Yes	Yes	Yes

**Table 3:** Characteristics of interventions evaluated in publications targeting persons with musculoskeletal disorders. Abbreviations: LBP: low back pain; MSD: musculoskeletal disorders. Empty cells mean either that the intervention did not focus on the aspect or that it was not described in the publication.

First Author Year	Country	Targeted Population	Strategy (Name)	Strategy (Description)	Multidisciplinary	Interagency Collaboration	Individualized	Early Intervention	Education Participants	Education Others	Self-Management	Workplace or Employer Involved	PTSL Allowed	Data Support Effect on Outcomes
Du Bois M. 2012 [32]	Belgium	Employees, sick listed (> one month) with LBP	Information and advice	Information and advice to stay active by medical advisers after a disability evaluation.				Yes	Yes		Yes			Yes
Jensen L.D. 2012 [33]	Denmark	Employed LBP patients expressing concerns about the ability to maintain their current job	Counselling	Counselling by an occupational physician addressing experienced workplace barriers and physical activity.			Yes	Yes	Yes	Yes	Yes	Yes		Unclear
Vermeulen S. 2011 [31]	The Netherlands	Unemployed and temporary agency workers; back, neck, other pain	Multidisciplinary intervention	Stepwise communication process to identify and solve obstacles for return to work, resulting in a consensus-based plan. The role of the return to work coordinator is to stimulate a high degree of involvement of both the sick-listed worker and the labour expert. A vocational rehabilitation agency was contracted to find a suitable workplace matching with the RTW plan.	Yes	Yes	Yes	Yes		Yes		Yes	Yes	Yes
Jensen C. 2011 Jensen C. 2012 Stapelfeldt C.M. 2011 [28,29,30]	Denmark	Employees, sick listed with LBP for 3 to 16 weeks	Multidisciplinary intervention	Examination by a rehabilitation doctor and a physiotherapist and reassuring explanations. A case manager conducts a comprehensive interview and designs a tailored rehabilitation plan to be discussed in the multidisciplinary team; the case manager contacts the work place and the municipal job centre to discuss and coordinate initiatives.	Yes	Yes	Yes	Yes	Yes		Yes	Yes	Yes	No(Jensen C. 2011) Yes, per group (Jensen C. 2012 and Stapelfeldt C.M. 2011)
Viikari-Juntura E. 2012 [37]	Finland	Employed, sick-listed with MSDs	Part-time sick leave	Reduced work hours with task modification, if required.			Yes	Yes				Yes	Yes	Unclear
Shiri R. 2011 [36]	Finland	Employed; seeking help for upper-extremity pain (different diagnoses)	Ergonomic intervention	Ergonomic intervention. After the clinical examination, the physician contacts the employer, and a visit by the occupational physiotherapist is scheduled. The workplace is assessed and possible accommodations discussed with the employee and supervisor.	Yes		Yes	Yes	Yes	Yes		Yes	Yes	Unclear
Myhre K. 2014 Marchand G.H. 2015 [34,35]	Norway	Employed neck and back pain patients, sick-listed for 1 to 12 months and referred to secondary care	Work-focused rehabilitation	A case worker analyses together with the patient work and RTW difficulties; they develop a RTW schedule; they and discuss relevant issues for a meeting with the employer; if sick-leave compensation is an issue, the caseworkers contact municipal social services.	Yes	Yes	Yes		Yes		Yes	Yes	Yes	No added value to multidisciplinary intervention (2014) Support for subgroup effects(2015)
Steiner A.A. 2013 [27]	Switzerland	Persons with chronic LBP, non-specific LBP with or without radiating leg pain	Multidisciplinary functional rehabilitation program	Sessions included: (1) cardiorespiratory fitness, muscular strength, muscular flexibility, stabilization exercises, relaxation, proprioception and water gymnastics; (2) occupational therapy with emphasis on individual professional and daily life situations; (3) patient education sessions based on a non-injury model and the biopsychosocial model; and (4) one hour per week of support group led by a psychiatrist. Personalized, realistic and measurable objectives were defined individually.	Yes		Yes		Yes		Yes	Intervention at the workplace not possible for all participants		Unclear

## Supplementary Materials

The following are available online at http://www.mdpi.com/1660-4601/15/3/552/s1, Search Strategies.
